# 

**Published:** 1882-02

**Authors:** 


### Independent Practitioner:

A MONTHLY JOURNAL,

DEVOTED TO

MEDICINE, SURGERY, OBSTETRICS, DENTISTRY, HYGIENE AND POPULAR SCIENCE.

EDITORIAL CORPS:

BASIL M. WILKERSON, D.D.S., M.D., EDITOR.

Associates:

GEORGE H. ROHÉ, M. D. GEO. W. FIELD, D. D. S.

NEW YORK:

*THE INDEPENDENT PRACTITIONER CO.,*

1099 BROADWAY.

**$3.00 Per Annum in Advance. - - - - Single Copies 30 Cents.**

*Entered at the Post Office, New York City, as Second-Class Matter.*

